# The 3Ps: A tool for coach observation

**DOI:** 10.3389/fspor.2022.1066378

**Published:** 2023-01-20

**Authors:** Jamie Taylor, Áine MacNamara, Dave Collins

**Affiliations:** ^1^ School of Health and Human Performance, Faculty of Science and Health, Dublin City University, Dublin, Ireland; ^2^ Grey Matters Performance Ltd., Stratford upon Avon, United Kingdom; ^3^ Moray House School of Education and Sport, The University of Edinburgh, Edinburgh, United Kingdom; ^4^ Insight SFI Centre for Data Analytics, Dublin City University, Glasnevin, Dublin, Ireland

**Keywords:** sport coaching, observation, coach development, PJDM, expertise, macrocognition, coach behaviour

## Abstract

There is growing recognition of the value of “*in situ*” coach development practice across a variety sporting contexts. Unfortunately, however, there remains a limited number of tools available with which to observe coaching practice. In this study, we pilot and test a quasi-systematic tool for observation in the form of the 3Ps. Drawing on a range of representational perspectives, the theoretically neutral labels of “procedure”, “planning”, and “process” were developed for the purpose of holistic observation. In order to test the tool, a group of experienced coach development practitioners (*n* = 10) integrated the tool into their practice over a 12-month programme of professional development. Those participants subsequently took part in semi-structured interviews, in which they expressed a strong sense of acceptability, perceiving effectiveness and positive opportunity cost. We propose that the 3Ps tool presents a holistic and practically useful means of observing coaches’ professional judgment and decision making. We also suggest future directions for the researcher who seeks to generate evidence in a naturalistic coaching context.

## 1. Introduction

Recent years have seen significant growth of the role of the coach developer (CD), with conceptual and practical advances highlighting the value of coaches receiving support to develop their practice (1). The term CD has often been used interchangeably with coach educator, mentor and, in some cases, tutor. More recently, the role has been defined as: “expert support practitioners who plan for, implement, and sustain strategies and interventions in support of skilled performance in sport coaching” (2, p. 4). This definition distinguishes the scope of the CD from other roles [e.g., coach mentor – (3)]; and highlights the more active, embedded, and complex pedagogic role of the CD. Notably, given the inclusion of “expert” in the definition, it is important to consider how best this process can be achieved.

One approach to the advancement of coaching practice which is advocated in the literature for is professional judgement and decision making [PJDM – (4, 5)]. PJDM is built on the foundation of an “it depends” approach to practice: recognising the inherent complexity of the biopsychosocial circumstances of coaching, and the need to take into account a wide range of contextual and individual demands when coaching (6). Although founded on a decision-making perspective, PJDM recognises the messy social world of coaching practice, in which multiple agendas and priorities influence a coach's practice (7). Crucially, the approach adopts a “for” coaching perspective: one that seeks to equip coaches with the resources to enhance their practice (8), rather than examining the process from a theoretical standpoint. This distinguishes PJDM from other approaches in the coaching sphere, which have aimed to describe what coaching is based on its ties to a particular epistemological or ontological orientation (9). So, rather than aiming to *model* the coaching process, PJDM instead offers a philosophy *for* practice, and suggests a practically oriented basis from which coaches can make decisions (10). From this foundation, researchers have identified coach decision-making as an area of research interest (11–13), and PJDM has been adopted as a model *for* practice in multiple domains – including forming the basis for coaching standards in high performance and coach development (2, 14).

Although sometimes misunderstood as a licence for practitioners to ignore research findings, PJDM is in fact the essence of research-informed practice – a concept more broadly accepted in learning and development (15). This recognises that tightly controlled experimental designs are unlikely to be possible, or generalisable, given the biopsychosocial complexity of coaching (e.g., 16). Operationalising PJDM relies on several core constructs (6), these include: engaging in nested planning with the bigger picture in mind (17); identifying intentions for impact (18); utilising declarative knowledge to support decision making (19); and adaptive intuitive practice, making adjustments that account for the broader whole (10). Specifically, when applied to the PJDM of the CD, there are a number of cognitive demands, including understanding of the context, the coach, adult learning, the curriculum, process and practice, and of self (20). Therefore, PJDM does not consider coaching, or coach development, to simply be a cognitive process. It draws on multiple perspectives in order to enhance the coach's ability to act in the messy social world (cf. 21).

### 1.1. Observing coaching practice

In order to optimise the process of coach learning, there has been increasing emphasis on “*in situ*” support, with CDs observing coaching practice alongside offering developmental input for the coach. Behavioural approaches have long been used for the interrogation of coaching practice, including early work observing the practices of John Wooden (22) and use of the Coaching Behaviour Assessment System (23). Subsequently, behavioural tools have been used both as a means of gathering empirical data to understand the work of the coach (24), and in practice for feedback to a coach. A more contemporary approach has been the Coach Analysis Intervention System [CAIS – (25)], proving popular both in research and in pockets of CD practice. The advantages of behavioural approaches have been emphasised because of its capacity to provide data on the work of a coach and how they interact with athletes (26). Similarly, behavioural analysis can act as a means of enhancing a coach's awareness of their actions (27), especially when a coach lacks the basic self-awareness necessary to engage in realistic discussions about their practice (28, 29).

Despite these advantages, however, several limitations also hold for behavioural analysis of coaching (30, 31). Although never intended to do so by behavioural analysis, behaviours cannot be used to predict effective coaching, nor do they offer a holistic view of coaching practice (32). This lack of holism presents a challenge for those looking to understand the relation between a coach's learning design, their coaching approach, and the experience of athletes (e.g., 33). Similarly, behavioural analysis has predominantly been used to observe coaches during training sessions (26) and, as such, it cannot cater to a fuller range of activities, especially when coaching practice extends beyond the track/field/court to a range of “off-field” settings (34). Naïve application has also seen behavioural analysis used to enforce “the right way” of coaching. Although strongly discouraged in the literature (35), it is something that remains a feature of the practical coaching discourse and an active discussion point among policy makers who seek to audit coaching practice. The limitations of this naïve approach have long been recognised, with the suggestion that behavioural analysis should instead be used to check for congruence against a coach's intentions (36).

More recent scholarship advocates for the use of dialogic pedagogy when interrogating a coach's practice (37). This dialogic approach may allow for researchers to utilise some of the tools that have been developed with the purpose of helping coaches to plan for and reflect on their practice, for example, the Coaching Planning, Practice and Reflective Framework (38, 39), and the “Big 5” structured approach to critical reflection (40). However, if these are employed as a means of generating feedback, there may be limited utility for the coach who is more fully aware of the pedagogical strategies that they are deploying (41). In short, whilst offering utility, behavioral observation behavioural observation remains subject to the critique that:

When the tasks that people are doing are complex, it is not enough to simply observe people's actions and behaviours—what they do. It is important to find out how they think, and what they know, how they organise and structure information and what they seek to understand better (42, p. 3).

For both practice and research, this leaves a choice of observational tools that all use behavioural analysis as a base, with all its acknowledged strengths and limitations. Until now, no alternative observational tool has been developed that supports the CD, coach, or researcher in critically observing practice through a PJDM lens. In essence, there is no approach that enables a view on the most fundamental feature of PJDM: the “why?” of practice (43). Therefore, this study had two overarching aims. First, to generate a tangible observation framework of coaching practice, grounded in a PJDM approach. Second, to explore the practical acceptability of the tool in an applied setting (acceptability being: “the extent to which people delivering or receiving [an] intervention consider it to be appropriate, based on anticipated or experienced cognitive and emotional responses to the intervention” (44, p. 4)). Specifically, we wanted to understand concurrent and retrospective acceptability for observing coaching practice. The focus for our research was therefore on whether practitioners understood the nature of the intervention, the opportunity cost compared to other approaches, and the perceived effectiveness and impact on the practitioner (44).

## 2. Method: piloting the tool

Drawing on the PJDM perspective, we aimed to develop a practical tool that offered utility to practitioners across a range of perspectives, without tying the CD to a specific decision-making paradigm. As such, the 3Ps (procedure, planning, and process) are theoretically neutral labels with which to categorise elements of a coach's practice. They were developed by drawing on a range of representational theories (cf. 45), including information processing (46), macrocognition (47), and literature from the active inference perspective (48).

Earlier conceptualisations of PJDM drew on both the dual systems approach to decision making and constructs from the macrocognitive paradigm (5). The dual systems perspective suggests that system 1 decisions are fast, frugal, and intuitive; with system 2 decisions being slower and more deliberative (49). Macrocognition offers a variety of cognitive functions used by practitioners including recognition primed decision making, sensemaking, projection, knowledge construction and flexecution (47). Between dual systems and macrocognitive perspectives, there is coherence and agreement on the core elements, with differences being more “emotional” than intellectual (50, p. 518). The key difference is that, by capturing a range of different cognitive processes, the macrocognitive perspective promotes a view that expert decision-making is not a reduction of bias or mistakes, but instead rests upon on discoveries, insights, and the use of mental models in action (51). Across both bodies of work, there is also recognition that faster, more intuitive processes are not distinct from more deliberate processes, but are interlinked, and that actions typically result from the relative weighting of both systems (52).

Since the development of the original PJDM work, there has been significant growth in the active inference paradigm, an approach with different epistemological roots (53). Active inference explicitly seeks not to challenge previous psychological frameworks, but instead to underpin key constructs from the psychological literature (54). Active inference suggests that all human behaviour follows the imperative of minimising the surprise of sensory observations with the active control of action–perception loops.^1^ Behaviour is theorised to result from deliberative and habitual processes, the contribution of each depending on the level of experience in context and relative investment of cognitive resources (54). Deliberative processes are those oriented towards the reduction of uncertainty for epistemic purposes, they are more versatile, but slower, and more costly (55). Habitual processes are those based on stimulus–response associations, considered to be fast, but inflexible, and promoted by increased experience in a specific context (56). Habit formation can also occur through the observation of goal-directed behaviour and the engagement of deliberative processes (57, 58).

At their root, these perspectives clearly offer different perspectives; but a degree of overlap would seem face valid (e.g., 50, 54). Any attempt to amalgamate different positions may lack coherence at their ontological roots; yet, from a pragmatic perspective, where one is interested in the “cash value” of knowledge (59), this overlap may be of value to practitioners (60). Thus, in real-world settings, we suggest that there is value in a multiple model approach (61). On this basis, we wanted to develop an approach that directly addressed the issue of moving beyond *what* a coach does, to an understanding of their decision-making processes, incorporating both deliberative and more intuitive decisions. As such, the 3Ps are not novel psychological constructs, but instead theoretically neutral labels representing the different processes that a coach might engage in. These, in turn, provide lenses through which a coaching environment can be observed, both in and out of session, allowing for inferences to be made of a coach's mental world. This acknowledges the inherent limitations of observational analysis and the subsequent interpretive role of the observer. It encourages observers to cast themselves in the mental world of the coach and athlete during observation, with the aim of better understanding a coach's practice. The Ps are defined, and examples of the underpinning theories for each element given, in Table 1.

**Table 1:** An overview of perspectives informing the 3Ps.

Element	Definition	Associated constructs and example references
Procedure	The basic competencies and administrational features of a coach's work that might be more easily reduced to “best practice” Not limited to: safety, organisation of the environment (e.g., time keeping, equipment organisation), management (e.g., positioning, voice projection, grouping of participants), and collective organisation with other coaches	Routine competencies (62) Coach behaviours (23) Performance of procedural skills (63)
Planning	The enactment of elements of practice underpinned by deliberative thinking, typically taking place offline, or during quieter periods. This would include engaging in nested planning and the process of re-planning	Deliberative processes (56) System 2 (64) Sensemaking (65, 66) Mental projection (67) Planning and re-planning (68) Nested planning (5)
Process	More time-pressured and intuitive features of practice, with the coach responding to affective concerns, changing situational demands or time pressure	Intuitive decision making (10, 69) Habitual processes (70) Perseverative processes (54) System 1 (64) Heuristics (71) Flexecution (72) Nested thinking (6) Recognition primed decision making – simple match (73) Tacit and experiential knowledge (19, 74)

The tool explicitly recognises the more competency-based and administrational elements of practice that could fairly be seen as “best practice”; that is, the basic competencies that could be identified as being demonstrably “good” or “bad” – termed here as “procedure”. “Planning” groups the slower, more deliberative elements of practice and expertise, while “Process” refers to the more intuitive elements that underpin adaptive progress towards intentions (75). Built into the approach is an acknowledgement that there is no arbitrary line between the constructs categorised as either process or planning. Indeed, any action taken by a coach is likely to have elements of faster, more intuitive elements, and slower, more deliberative underpinnings. This is explicitly recognised by all of the constructs that inform the 3Ps structure. We can, however, make useful generalisations about styles of cognition, which has also been the case in the literature (52). Categorisation is a matter for the professional judgement of the observer and should be subject to later triangulation. In addition, there is no arbitrary line to be drawn between a coach's approach on or off the dojo/court/pitch/track. For example, a feedback conversation with parents following a training session will rely on the same processes as those used during the session. In practice, this enables CDs to observe all elements of coaching, within and beyond the training or competition context.

### 2.1. Intervention and testing the 3Ps

Following the development of the framework, we wanted to pilot the use of the 3Ps tool, testing its utility and acceptability in coach development practice. Adopting a similar approach to one previously used to pilot coach development tools, the study was conducted in the context of an advanced CD training programme (40). A participant group of 10 experienced practitioners each opted into the development programme *via* their national sporting organisation (9 male and 1 female, M^age^ = 40.5, SD = 6.74), with a mean 21.4 years’ coaching experience (SD = 5.53), and a mean 11.8 years’ experience working to support coach learning (SD = 4.81). All participants held previous qualifications as a coach educator or mentor, eight held the highest coaching award available in their main sport, and seven held master's degrees in an area related to sports coaching. Participants in the sample were supporting coaches on the full spectrum of experience, from relative novice to highly experienced professional; and across the domains of children's participation, talent development, and performance (35). Given the limited population of coach development professionals, no further demographic information is offered in order to protect the anonymity of the participants.

Over the course of a 12-month programme of development, participants engaged in training to support their practice as CDs. This included support for broader elements of CD practice such as: understanding PJDM as an approach, various pedagogical approaches, and feedback/debrief processes. Specifically, it also offered an introduction to the 3Ps, their theoretical underpinning, and suggested means of practice from the author team. Part of this support advised CDs that observations could be conducted in multiple ways. For the purpose of the 3Ps, CDs were advised to apply a structure to their field notes, based on a split of three columns, one representing each category. In addition, CDs were advised to add a time stamp to each observation, enabling the capture of observations made prior to what would traditionally be considered “in session” or competition (e.g., conversations with athletes and other coaches prior to training). In order to support the multiple lens approach, CDs were advised to deliberately switch between different frames of reference during observation, in line with each “P”. It was recommended that CDs review these field notes following an observation, with the aid of any video or audio captured from the coaching environment. Concurrent with this “training”, CDs had the opportunity to embed the 3Ps in their practice. Support was offered by the first and second authors (JT and ÁM) acting as “meta coach developers”, with two “*in situ*” visits to observe the practice. These visits were supported by follow-up training interventions on other elements of CD practice, along with retrieval of initial ideas. Although by opting into the programme the participants could be considered as motivated to develop their professional practice, they were under no obligation to integrate the 3Ps in their work, nor deviate from their normal practice.

### 2.2. Data collection

Following protocol approval by the Dublin City University Ethics Committee, the first and second authors (JT and ÁM) conducted semi-structured interviews with the CD participants to understand their existing practice, concurrent and retrospective acceptability of the tool, and creative practice. A semi-structured interview guide was constructed in order to gauge each participant's understanding of the intervention, opportunity cost compared to other approaches, perceived effectiveness, and impact on self-efficacy of the practitioner. Specifically, we sought to understand (i) the CD's previous observational practice; (ii) if and how they had used the 3Ps; (iii) changes to their coaching observations; (iv) if they have made adaptations to the tool; and (v) perceived changes in their wider professional practice. Interview questions included: “Can you outline your historic approach to the observation of coaches?”, “If you have, how have you used the 3Ps in your practice?”, “Has your practice changed in anyway because of the 3Ps?”, “Have you, or do you foresee any advantageous adaptations of the tool?”, “What are the relative strengths and weaknesses of the tool?” Interviews were conducted following the completion of the wider development programme, in order to reduce any perceived need for impression management. It was made clear to participants that they were in no way obliged to take part in the research as part of the development programme. However, the richness of interview data was enhanced by the history of interaction between researchers and participants; and the accompanying understanding of professional practice and their social context. All interviews were conducted using video-conferencing technology (Zoom Video Communications, San Jose, CA, USA, Version 5.7) at a time to suit the participants. The interviews were audio-recorded and subsequently transcribed verbatim.

### 2.3. Data analysis

Due to the relative novelty of the subject, a deductive and then inductive content analysis was conducted to examine the acceptability of the 3Ps. This process reflected the assertion of Braun and Clark (76) that coding and data analysis involves a combination of processes. The deductive phase enabled the identification of information to address the research question; the inductive phase ensured the analysis was open-ended and that respondent-meaning was emphasised. Data analysis using QSR NVivo software took place in five steps: (1) Following transcription, the transcripts were read and re-read for familiarisation. This enabled the first author (JT) to become more familiar with the content, with notable material being highlighted and annotations made. (2) In this next step, quotations were deductively tagged in relation to (a) whether the 3Ps was a tangible observation framework of coaching practice, grounded in a PJDM approach, and (b) the extent to which a group of experienced CD practitioners accepted the tool. (3) Following this, a thorough inductive content analysis was performed, which involved moving recursively between open coding, focused coding and organising categories into higher order themes. (4) Constant comparison across different participant interviews and critical reflection was used to guide this analytical process (77, 78).

### 2.4. Trustworthiness

As is recommended by Nowell and colleagues (79), there is a need to demonstrate that qualitative research is conducted methodically and rigorously. In doing so, trustworthiness can be achieved through establishing credibility, transferability, dependability, confirmability, an audit trail, and deploying a reflexive approach (79). This led to the second author (ÁM), another experienced qualitative researcher, acting as a theoretical sounding board to encourage reflection and consideration of the broader dataset (80). The third author (DC) also acted as an overall critical friend, challenging broader theme-generation (81). In addition, the first author (JT) maintained a reflexive journal for the purpose of reflecting on the power dynamics during data collection, and the role of the researcher in shaping knowledge-generation. The diary also served as an audit trail, documenting methodological steps, the interpretation of data, and managing the analysis process (79). We ask the reader to judge the credibility and transferability of findings, based on the significant use of participant data and the thick descriptions presented in the results.

## 3. Results

Results are represented by six themes, supported by direct quotes from CDs throughout. CDs described their previous practice, the impact on their “*in situ*” practice, the impact on broader professional practice, the perceived advantages and disadvantages, and any innovative use of the tool.

### 3.1. Previous practice

CDs described the use of three predominant approaches to their practice: the use of competency checklists, structured behavioural observation, and unstructured general observations (Table 2). CDs reported a general sense of dissatisfaction with these approaches, with a feeling that no single observational tool offered them what they needed to enhance coaching practice. For example, some CDs reported the naïve use of behavioural tools: “we had behaviours that we look for, you know, questioning and letting players explore. I would count up how many times they used good behaviours and bad ones” (CD10).

**Table 2:** Historic use of observational strategies by CDs.

		Raw data examples	Number of CDs reporting as a feature of practice
Previous observation practice	Competency-based approaches	“It was a tick box exercise to see if they’re at the right standard. I think when it's competency based, whenever we’re looking everything was steering towards an answer that you had to find. We almost try to fit a coach to a set criteria, rather than looking at what is going on” (CD1) “Essentially, I used the form given to us by [NGB] which was a tick box to evidence what they’ve done, or not done and then a comments box” (CD7)	5
	Behavioural strategies	“I would have been very behavioural, tagging coaching behaviours and then just providing feedback on that” (CD9)	4
	General observation	“For me it was just notes, there was no structure no real, I looked at what the coach was most interested in and I took notes against that” (CD4) “It wasn’t working off a model, it was observing what was happening in the session chronologically. Trying to help the coach generate some feedback from that process …. How long did you spend on demonstrations? How long did you spend on each section? It might have been around athlete behaviour or around interaction” (CD6)	4

Given the experience of some of the CD group, a number reflected on the changing nature of their practice over time. As a result, some had used various approaches over the time that they had been a practising coach educator or developer. For example, CD3:

I’ve been around long enough to go through different processes. Initially, it was a tick box based around what the coach did and how they did it, the coach had to evidence being competent within each stage to get a tick. That process has changed over the last three four years to using a behaviour template.

### 3.2. Impact on “*in situ*” practice

For CDs, the 3Ps were utilised in three predominant ways: first, as a tool for structuring their observation of coaching practice; second, as a means to inform their feedback, or debrief processes; and third, as a frame through which to enhance their overall sensemaking of the coach's unique circumstances. These themes are presented in Table 3.

**Table 3:** Impact of 3Ps on “*in situ*” practice.

		Raw data examples
Impact of 3Ps on “*in situ*” practice	Structuring observation	“It's very different to just watching what happens. It is about understanding why they are doing something, or why not?” (CD4) “I’ve got more rigour and coherence, but that's through structure, it's just a much better platform that I didn’t have before … the 3P's really allows you to structure things” (CD6) “More rigor because it because of the way I think it gives a very wide lens. I see a broader range of things than with other approaches. It has changed me overall; I look more broadly and not just at what pops up in the session” (CD9)
	Sensemaking	“There is a big element of judgment and your own opinion on what you observed. I suppose you need to balance that up in terms of what's what can make the most impact on the coach on their coaching. My last observation … I had three pages of notes with maybe 10 areas that we could have considered, but I used it [3Ps] to reflect and narrow things down” (CD2) “I use it as a bit of a framework to firstly think about what I was observing within the session and consider the needs of the coach. With [coach name] their planning was really strong, what their aims are, how they were going to achieve their aims. Procedurally, it was smooth, all the logistics and everything. But, in the actual session, they couldn’t adapt to a couple of changes and so couldn’t work towards their aims” (CD5)
	Informing post-observation learning strategies	“First of all, it's a really useful way to capture what I’m observing. Secondly, it's a really useful way to feedback or structure the conversation with the coach” (CD6)

#### 3.2.1. Structuring observation

In the case of acting as a tool for structured observation, CDs placed a high value on the use of the 3Ps:

It is really effective for structured observations and will give me most of the things that I needed from the session and the coach's environment … that might be why it has been so well received; it's made a big difference to our practice (CD4).

The 3Ps allowed CDs to deliberately change frames of reference in observation, for example, CD7 suggested that: “rather than trying to look at everything, it really helps me organise what I am looking at, it helps my mind be more organised, almost like drawers to open and think a different way”. A significant part of this focus allowed for the observer to consider how a coach's intentions and mental processes were influencing practice. The deliberate switching of lenses seemed to characterise CD's adoption of the tool, seeking to understand the same events, but from different perspectives. It also seemed to support CD's metacognition and monitoring of what they were attending to:

It allows you to think “why did I see that?” Why did the coach ask that question instead of doing it this way? And ultimately, I think this really helps me to impact the coach later (CD2).

Similarly, CD9 described how the structure influenced metacognitive monitoring of their own approach:

The biggest thing is that I’m zoomed out for longer. Before I’ve been inclined to pay attention to one thing. Now, committing less to an area when it pops up in the session, but also able to look at the whole. I guess, a zoomed in and zoomed out approach isn’t it. I look at the full detail and breadth of what's happening. It helps me get into a specific part of the session, or much broader, into the whole environment.

It was this attention to the wider coaching environment that was perceived to be of significant benefit, with the CD not only observing within a session, but observing more broadly and considering the wider context of the coaching environment: “the structure really helps me, not just in the session, but also the rest of the environment” (CD10). This process in turn, seemingly promoted a holistic means of observing coaching practice.

#### 3.2.2. Sensemaking

The breadth of the 3Ps structure allowed for a wide range of data to be collected from observations. Along with in-observation sensemaking, prompted by the nature of the categorisation process, post-observation sensemaking was supported. The tool's structure allowed for a review, both of notes taken and of video footage of coaching observations:

I revisit my notes and the video afterwards. They [3Ps] have allowed me to think again about what I saw, to really ask the question of why things are happening and I’m taking time to do that … I had this really difficult session with a coach, I didn’t know where to start because there were so many issues. The 3Ps enabled me to get the coach thinking about specific areas without me really having to go any deeper until I had the time to reflect and debrief for myself (CD2).

This reflection and sensemaking also took place in slower time, considering the needs of the coach and where the session fitted in the broader context. This was often described by CDs as a process of deciding which data to use with the coach given the volume of potential avenues for further investigation: “I have to narrow things down to make sure they get some sort of coherence from me” (CD8).

#### 3.2.3. Informing post-observation learning strategies

The third use of the 3Ps tool was in crafting CDs' pedagogic approach for the coach in the immediate follow up after an observation, with the framework directly informing their feedback or debrief processes. This allowed CDs to steer conversations with coaches, keeping a bandwidth on the number of focal points for reflection, and to debrief with good judgement: “previously, I was being a bit too judgmental, and giving my own opinion, whereas now, I’ve got options in the debrief” (CD2). In some cases, CDs chose to explain the tool to the coaches that they worked with:

I’ve used it to help structure feedback. Rather than bouncing all over the place, I have explained to them and asked them to consider their thought process, almost auditing what I saw. This bit of debrief gets them reflecting and we can then look at the concepts that I have observed (CD6).

Explaining the framework to their coaches enabled greater understanding and aided the coach's reflective processes.

After working with a coach for a while, I asked them to go through the 3Ps themselves when they were reviewing a session. They used the framework, watching it [the session] back and making notes for discussion (CD10).

### 3.3. Impact on professional practice

In addition to shaping their practice with the coach, using the 3Ps also seemed to influence CDs' broader practice, encouraging them to approach the development of coaches in subtly different ways. CDs described other impacts on their work, indeed shifts in their professional philosophy as a whole, and how the tool impacted their perceptions of their role. These themes are presented in Table 4.

**Table 4:** Impacts on professional practice.

		Raw data examples
Impact on professional practice	Informing PJDM of the coach	“It's allowed me to put myself in the shoes of the coach. I can see more of what they’re trying to do, how they adapt and how do they deal with it” (CD5) “I look for meaning so much more now. What is the meaning of this particular event, rather than just writing it down. I am putting myself in the world of the coach so much more” (CD9)
	Focus on longer term impact for the coach	“I’m looking beyond the actual session now. Going deeper and thinking about the big picture for the coach. I am thinking about what else is going on” (CD1) “I went from a change of behaviour focus to a change of thinking for changing behaviour over the long term” (CD10)
	Informing CD PJDM	“I’m working with two coaches; they’re actually partners in life as well. I’ve taken two completely different approaches with each of them based on 3Ps … they are at very different stages in their careers. I can go into the club, watch each of them and help them both individually using the same framework for observing” (CD8)

#### 3.3.1. PJDM of the coach

The first and most prominent area of change for CDs was the shift from observing only what a coach did, to observing with an interest in why the coach was taking the actions that they were:

It's [the 3Ps] way more contextually relevant, and more personally relevant to the coach. I’m really trying to understand why they’re doing what they’re doing, using my understanding of the coach and their circumstances (CD7).

The consequence of the focus on the PJDM of the coach allowed for the CD to support the development of a coach's knowledge in their context and not in the abstract:

It has significantly impacted the value I put on hearing from the coach, treating them as a person and a professional. It is now about working with their embedded knowledge, helping them to reflect on their practice and their beliefs. It's changed my view away from just thinking about best practice (CD8).

This shift towards a more contextualised understanding of the coach and their work seemed to give the CDs a greater insight and a perception that they were better able to support individuals' learning and development.

#### 3.3.2. Focus on longer term impact for the coach

The second theme concerning impact on the CDs' work was the view that using the 3Ps enhanced the ability of CDs to see beyond the confines of a specific session and identify areas of impact for the coach. This was partly a result of putting themselves in the mental world of the coach and using data captured to consider the broader impact on the coach's learning and development. For example, CD8:

It has helped me move from being very procedural in my practice, to a focus on the “why?”. The epistemic stuff. I was at a training session with a coach last night. It was very structured, very drill based. There's nothing inherently wrong with that, but it was clear the girls were just going through a practice without any purpose…. We had a really good conversation afterwards about his reasoning, it really helped me get into and understand his beliefs (CD8).

This necessitated a change from offering a personal perspective on what the CD would do if they were the coach, to an interest in longer-term impact, helping the coach to reflect on their work based on longer-term needs: “previously I observed and just gave my opinion. Using the 3Ps, it focuses me a wee bit more on the bigger picture” (CD2).

#### 3.3.3. Informing PJDM of the coach developer

The final change in practice concerns the shift in the PJDM of the CD. In all cases, CDs described a sense of being better-equipped to support coaches and being more informed in the decisions that they took to support the coach. For example, in the case of CD4:

It informs me formulating logical steps. I am thinking two, three, four steps ahead. Those three, four steps ahead are in the back of my head, I'm thinking: “how is the coach going to react?”. Which bit do I generate feedback on first? (CD4).

CDs expanded on this view and described the feeling that it enabled them to meet the needs of more challenging coach cases:

With the tricky ones [coaches] I always used to get stuck thinking “where do you start?” This can take you as deep as you need to go. It really helps with my process … whilst the 3Ps might never be spoken about between me and the coach (CD4).

CDs described using greater degrees of flexibility and creativity in their practice, using the levels of data generated to meet the bespoke needs of the coach. This was especially the case for those CDs who had previously either relied on competency-based approaches, or naïve interpretations of behavioural observation: “it invites a more flexible approach from me. I can use different tools afterwards. It isn’t just: ‘you did this, you did that’, or ‘you didn’t tick this box’. It gives you options” (CD3). Part of this option-generation came from CDs using the 3Ps to influence their pedagogic process following the observation. This typically took the form of structuring feedback or debriefing processes immediately following observations:

I feel it helps with my approach as a CD. I really value practical, intimate coaching conversations with the coach. It is potentially my bias, but I feel this [3Ps] helps me do it. I can have a coherent flow to my work (CD6).

This, in turn, seemed to influence a consideration of options: “I’m much more confident providing or generating feedback or debriefing effectively. This [3Ps] gives me options and coherent routes to follow, I have really genuine options” (CD1). In essence, it seemed to offer possibilities for informing an appropriate pedagogic strategy, whether guiding a coach through a debrief, offering feedback, or generating feedback.

### 3.4. Perceived opportunity cost of the tool

As a feature of the pilot, CDs were asked to consider what they considered the opportunity cost of the 3Ps to be, compared with other coach observation tools they had experience of (as outlined in Table 5). As identified earlier, the approaches that CDs were aware of included competency checklists and various forms of behavioural observation.

**Table 5:** Perceived opportunity cost of the 3Ps.

	Perceived Advantages	Perceived Disadvantages
Relative to competency approaches	“With a competency-based approach, it's superficial. It's not adaptive to what's happening within the environment, it's not adaptive to the needs of the culture. It's just generic” (CD5) “By virtue of the fact that it is just a checklist is the limitation. There is no qualitative aspect to it. It was just a simple yes or no. They’ve evidenced something: ‘yes or no’. Whereas with the 3Ps it is the starting point” (CD7)	“I’d say competency-based frameworks, look good on paper for governing bodies, they think they can just standardise for everyone, but that isn’t the real world. Whereas with the three P's you can offer a more holistic approach, I think it's a lot more complex and requires more knowledge and skill from the coach developer” (CD5)
Relative to behavioural observation	“It's different to just counting. If we can get to the why, then I understand a lot more, and there's a lot more depth, and there's a lot more context to, you know, what they’re doing and how they’re doing it” (CD6) “It [3Ps] allowed me to get a broader view of the session. It might be a simple structure, but it really captures the complexity of what's going on … By the end of sessions, I am walking away thinking in a more structured way. It enables us to code what we are looking at, for the coach that's advantageous to me because it allowed me to identify sort of key themes that were emerging. I felt like it's a more human way” (CD4)	“I think a lot of the coaches we work with know what they’re doing, which is great. Sometimes they don’t, they know how to do it, but they genuinely can’t see what they are actually doing. For that, I think the 3Ps sometimes works, other times, you need to smack them round the face with numbers a bit” (CD3) “Some coaches I’ve worked with really didn’t really know what they did. I’m using behavioural analysis a lot less now, but with coaches that have no idea what they actually did, I think it's useful for a one off … Behaviours might be useful as a starter if they’ve got no understanding, or if they are closed off. From there, the 3Ps are so much more versatile and useful” (CD1)

In comparison with a competency-based approach, there was a strong perception that the 3Ps tool offered significantly more for CD practice. The key advantages being described as the adaptability of the tool and its ability to meet the needs of the coach and their context. One negative point that was identified was the potential for governing bodies to prefer a universal standard against which to assess every coach, regardless of age or stage. Another negative was the perception that the 3Ps tool is not something that could be used without a level of expertise.

A more nuanced view of behavioural approaches was offered. Behavioural observation was discussed by CDs as a different tool, for a different purpose. In some cases, CDs described naïve applications of behavioural analysis as a contrast. Though, in comparison with less naïve behavioural approaches, CDs welcomed the flexibility and holism that the 3Ps offered. The tool also appeared to be favoured for its ability to generate a broader range of data, both across the environment, beyond what the coach did, and understanding why a coach chose an approach. CDs also described being able to move beyond observing a coach's behaviour alone, to considering their learning design and the technical/tactical components of their coaching: “It helps me go beyond coach behaviours. It gets into other areas of coaching, the technical, tactical and their practice design and what's going on with the athletes” (CD10). There was however a perceived disadvantage to the use of the 3Ps tool when working with coaches who lacked self-awareness in their approach and who were resistant to the input of the CD. In this regard, it was perceived that the more “objective” data generated by systematic behavioural analysis might be useful for offering feedback to a coach.

### 3.5. Innovative use of the tool

The final area of interest in piloting the tool was to understand if the 3Ps had been used in creative or innovative ways. Most CDs suggested no significant adjustments to the tool. Where CDs had innovated or foresaw changes in their use of the tool these were additive, rather than adjusting the structure of the tool. As an example, CD1, who had experience of behavioural observation, discussed a blend with the 3Ps for coaches who lacked self-awareness or saw their practice paradigmatically differently to the CD:

If they are miles off in their self-awareness, I might start using the 3Ps with some behaviour tracking. I might run both at the same time, as long as I’ve got video, but I think I will always start with the 3Ps and use behaviours in the procedural bracket if I have video to refer back to (CD1).

This additive combination was also noted by CD9 who suggested: “if there is a clear need, I have used other frameworks like the Coach Planning and Reflection framework. But probably only when the focus has been narrowed down a little bit”. Others suggested a potential expansion of the tool, allowing for a more fine-grained analysis of observations. This was proposed as a means of either contributing to observation at the time, or, for greater depth, utilising video footage for slower analysis: “you could have subsections in each of the factors. So, you could have macro, meso, micro aspects of planning. For procedure, you could have subsections to analyse coaching behaviour” (CD6).

Other innovations included the narrow use of the tool to focus entirely on a particular area of practice based on previously identified needs, and how this might influence what a CD may observe: “I might just take one of the elements, for example planning to generate feedback. It changes the emphasis on what you might want to observe, like a planning meeting” (CD9). Others suggested that after working with a coach for a while, they might ask them to observe the session using the approach, both as a means for generating a different type of feedback and also as a check for coherence between the CD and the coach: “after working with a coach for a while, I can see myself asking the coach to go through the 3Ps themselves when they are reviewing the session. Watching it back and making notes for discussion” (CD10).

## 4. Discussion

The purpose of this study was to pilot a research-informed tool for the purpose of coach observation, testing its utility and acceptability in CD practice. Findings support the practical utility and acceptability of the tool in a small cohort of experienced CD practitioners. In all cases, the tool was perceived as highly acceptable by participant CDs, who discussed changes to their professional practice as a result of the trial. We have also identified significant strengths of the tool, with it seemingly offering a more holistic and flexible view of coaching practice. In addition, with a pragmatic orientation in mind, we aimed to understand the opportunity cost of the tool in applied practice, relative to other observation approaches.

### 4.1. Professional judgement and decision making (PJDM)

Aligned with the purpose of the tool, there was a strong view that it enhanced CDs’ ability to enter the mental world of the coach and make inferences about the cognition behind their action. This seemed to enable CDs to move beyond what they were seeing and support active sensemaking, forming tentative hypotheses for coach decision making (66). As a result, we would suggest that the tool allows CDs to observe and infer coach decision making on multiple levels. It appears that the 3Ps may enable a fuller understanding of a coach's needs. While it may be less useful for coaches with low self-awareness of their practice, or those who are less open (82), the perception of utility held for coaches across a spectrum of practice from beginner to expert. Where the emphasis for a beginner coach might sit more at the procedural end, developing basic competencies, the focus for more experienced coaches might be more on the intuitive or deliberative elements of coaching expertise (83). Therefore, in addition to observation, CDs felt that the tool offered a frame with which to make sense of the needs of the coach, along with informing the pedagogic strategies that they themselves might deploy following the observation (65). As such, the 3Ps tool could inform a flexible approach to CD practice, where professional judgments could be made regarding the data used to offer, or generate, feedback. In essence, appropriate use of the tool seemed to inform how the CD actively shaped the learning experiences of coaches (84). CDs also believed that the tool had a broader impact on their professional practice than “*in situ*” observation of coaches alone. There was a sense that use of the tool enhanced professional effectiveness by helping the CD's to focus on reasoning strategies and underlying belief (85). This focus was perceived to help CDs engage coaches in more transformative reflective practice (cf. 86).

### 4.2. Holism

In addition to this perception of improved effectiveness, CDs also described a greater sense of holism, taking the view that the 3Ps encouraged a greater breadth of observation. This allowed for the capture of a coach's practice beyond discrete actions. The 3Ps tool may encourage a broader view, considering the meaning behind a coach's approach. There was also a sense that it enabled observation beyond the confines of a training session or competition, into wider coaching settings, for example classroom sessions (87) or “offline” work with other coaches and staff (17). It also allowed CDs to consider the nuanced interpersonal dimensions of coaching practice, for example, why a coach might be approaching a given interaction with a particular affective tone (88). That is, the tool offers the potential for the CD to infer the social intuition of the coach, the “rapid and automatic evaluation of another person's cognitive and/or affective state” (69, p. 308). In essence, allowing CDs to quasi-systematically observe a coach's learning design (89), their pedagogic approach (90), interpersonal dynamics (91), and infer the experience of athletes (33). Parallel consideration of these interlinking constructs presents the ability to reflect on these observations through multiple lenses, including the pedagogic, various “ologies”, and, where appropriate, the technical and tactical (19). As an example, the recent move to conceptualise feedback as a process, rather than something the coach does (cf. 92, 93).

This holism would seem to be a particular advantage for moving forward from the conceptualisation of coaching being something that the coach does, to a bi-directional, or indeed multi-directional, process. The 3Ps tool can offer a more holistic view of coaching, taking account of domains that previously might not have been structured elements of the CD's observation, and so offering an additional dimension to CD practice and research. Of all these elements, it is the technical/tactical dimension that has not traditionally formed part of CD practice. To be clear, we make no suggestion that the tool fundamentally changes the CD role frame. Instead, that the tool offers significant flexibility depending on the expertise of the CD and the nature of the CD–coach contract.

### 4.3. A tool for practice

The genesis of the 3Ps tool was a matter of practicality, aiming to offer a research-informed approach to the observation of practice primarily for the use of CDs. In considering the opportunity cost of using the tool CDs, while emphasising the broader utility of the tool, noted the challenge of using the 3Ps with a coach who lacked any self-awareness in their practice (29). This may be an issue if optimal impact with a coach is seeing a disparity between what they think they did and what they did. For the CD, it may be the case that using tools like the CAIS (25) and subsequent quantitative feedback may prove useful in moderating this tendency (29). Importantly, in no cases was the use of a competency-based approach identified as having advantages over the 3Ps, with the exception that it may provide a false comfort of apparent standardisation for national governing bodies.

For the CD, there is a need to see where and when various tools are most appropriate, and how they might inform observational practice. In both cases, it is important to note that CDs should use the 3Ps in a qualitatively different manner, for different purposes than other observational approaches. As noted by one of the participants, the 3Ps should be used as a window into the PJDM of the coach, informing the future direction of CD practice. As an observational tool, it can provide a range of data with which to tackle the cognitive demands faced by the CD (20). Given the context of many coaching settings, the 3Ps may also enhance the observation of whole coaching teams, rather than just the individual coach. The tool offers the flexibility of multiple lenses on the coaching process, allowing the observer to deliberately adopt different foci. Finally, there is the question of how much support CDs require in order to use the tool – as highlighted, it appears to require a significant depth and breadth of knowledge on the behalf of the observer (19). In practice, the 3Ps cannot be used as a formula (cf. 94), effective use will rely on the breadth of knowledge and expertise identified as a minimum standard for effective CD practice (2).

### 4.4. A tool for research

In addition to practical use, we suggest that the 3Ps tool also presents significant potential for evidence-generation in coaching. As a naturalistic research tool, it is not designed to provide the type of evidence emanating from tightly controlled experimental designs (68). As such, for the purpose of research, the approach should not be seen as a replacement for behavioural observation, which offers a more controlled and systematic, but less holistic, approach. The 3Ps tool actively recognises the interpretive role played by the observer in aiming to understand the mental world of the coach and the broader context. In this regard, if the aim is to understand the “why” of practice there is a need to understand what coaches are thinking, their knowledge, and how they make sense of events (31). Building on the reflections of Gallimore and Tharp (95) who suggest coupling systematic observation with qualitative methods for a richer account of coaching practice, future CD and research practice may wish to couple the 3Ps with other methods as a means of triangulation and “thickening” data. For the CD, this may be as simple as engaging in reflective questioning with a coach (37, 40, 86). However, for the researcher, aiming to address the long-identified lack of evidence from real-world settings (35) could involve triangulation using cognitive task analysis tools (96). Specifically, tools such as the critical decision method (97) could be coupled with observation to deepen insight. Depending on the style of research, or CD work, it may be appropriate for various combinations and adaptations of CTA tools to be used – for example, a critical decision audit to understand the coach's perspective on observed practice and the knowledge sources that underpin their work (98). This points to the opportunities presented by the various tools that have been developed for knowledge elicitation and how they might be combined with observations of coaching practice (99). In short, there appear several potential future applications of the 3Ps tool for both research and practice, each offering a fuller capture of a coach's naturalistic cognition and their practice.

### 4.5. Limitations

It should of course be recognised that there are a number of limitations presented by this study. First, although there are several advantages conferred by established relationships in qualitative research (100), it is important to consider the potential for impression-management influencing data collection (101). Deliberate steps were taken to mitigate this with interviews timed to take place after the conclusion of the CD development course and the programme review. To further combat this, we sought the views of participant CDs on the relative utility of the approach, against other common approaches, in accordance with the notion of acceptability (44). It is also important to acknowledge that the participants using the tool engaged in professional development that enabled them to build the declarative knowledge necessary to use the tool in practice. As such, it is unlikely that practitioners or researchers who wish to simply pick up and use the tool without this declarative understanding will have the same experience. Therefore, although the universal support for the 3Ps offers clear evidence for the acceptability of the tool, it may be the case that further research and validation among other populations are necessary. Finally, the potential for confirmation bias on the behalf of the research team is clear – for this reason, thick participant data is used throughout the results section to enhance credibility (79).

## 5. Conclusion

This paper has presented a novel approach to the observation of coaching practice with an emphasis on the PJDM of the coach. Findings suggest that the 3Ps tool may provide an opportunity to expand our understanding of PJDM in practice (5, 6) and support a base of evidence in practice that accounts for the context of particular approaches. While not suggesting that coaching is solely a matter of individual cognition, the framework was designed as a pragmatic tool to support the observation of coaching practice and make inferences about the PJDM of the coach. The tool was generated through an evidence-informed approach, drawing together multiple strands of representational research and professional practice. From a cohort of experienced CD practitioners, who had previously engaged in a 12-month programme of professional development, the tool received universal support for its acceptability, with high levels of understanding, positive reflections on opportunity cost relative to other approaches, and effectiveness of approach. We therefore suggest that the 3Ps may become a useful feature of the CD's and researcher's work, offering a tool with which to observe practice and support the development of expertise (102).

## Data Availability

The datasets presented in this article are not readily available in order to protect the anonymity of participants. Requests to access the datasets should be directed to jamie.taylor@dcu.ie.

## Footnotes

^1^For a more detailed exploration, we refer the reader to Linson *et al*. (53).
